# A Very Creditable Case—Life Saved by the Doctor

**Published:** 1855-09

**Authors:** E. Harris


### A VERY CREDITABLE CASE—LIFE SAVED BY THE DOCTOR.

BY E. HARRIS, M. D.

Margaret——, a chambermaid, æt. 20, residing at No.— Fourth Avenue, retired to bed about 11, P. M., in a dormitory six by ten feet, very closely finished, and furnished with a gas-burner having an unshouldered or freely turning stopper. She extinguished the gas-light in the usual manner but was so unfortunate as to turn the stopper 180 instead of 90 of the circle, thus permitting the gas to escape in a full stream. She fell asleep immediately; and at six o'clock the following morning, making no response to calls at her door, and the strong odor of gas arousing suspicion, the room was entered from the street window. The poor girl appeared utterly lifeless. I saw her at quarter past six. There was no perceptible respiration; no pulsation could be felt at the wrist or in the carotids: pulsation of the heart was barely observed by auscultation; only one movement was heard, and that so feeble that it seemed to be the last flicker of life. The surface of the body was cool, the skin maculated and purple, tongue and mouth cold, and the lips livid. The eyes were open, and the pupils were dilated to the fullest extent.

While the foregoing brief examinations were being made, cold water was procured and dashed upon her head, face and chest; carb. ammonia and brandy were also freely administered by the rectum.

The third or fourth dash of cold water upon her head and chest induced a short gasp and sigh: and at each succeeding dash, this pleasing evidence of returning animation was repeated. Occasionally, these gasps were succeeded by two or three scarcely perceptible respiratory movements; but it was not until the expiration of *three hours* that there occurred a full and natural respiratory act. It was not until the last of the *fourth hour*, at 10, A. M., that *regular* respiration became established, and continued without the aid of the reflex excitant, or water dashed upon the face. The heart at that time was pulsating at the rate of about ten per minute, and a feeble pulse at the wrist.

Sinapism, aqua ammonia, and artificial heat, were freely used from the beginning, and the administration of carb. ammonia, continued.—Other means were resorted to, but without any marked effect, except, perhaps, a strong infusion of coffee, which was given by way of experiment, from its known influence upon the brain and nervous system. The influence of this upon the brain was obviously beneficial, yet not so marked as in cases of poisoning by opium. Sulphuric ether exerted but a very transiently beneficial effect. It roused the circulation for a moment, but the cerebral functions seemed impaired by it. A strong current of fresh air directly upon the face, with friction and general excitants to the reflex system, were the only remedial agencies relied upon. Indeed, after regular respiratory action became established, pure air was the only remedial agent required. But during the whole period from 6 until 10 o'clock, it required the greatest watchfulness and activity to keep up an imperfect, gasping respiration.

Sense of consciousness began to appear at 2, P. M., eight hours from the time she was first seen in the morning. The eye began to exhibit susceptibility to the light at 1, P. M. The heart began to beat at the rate of thirty per minute. From this time, the condition of the patient steadily improved until 6, P. M., when she was able to converse rationally, sat up, and took nourishment. About 1 o'clock, the head became excessively hot, and the pupil became contracted. An ice cap and pillow were then resorted to, and kept closely applied to the head most of the time during the afternoon. The breath continued to have the strong odor of gas until 8 P. M., and the same was true of the perspiration and the renal secretion.

The girl slept well the succeeding night, and no symptom of any cerebral or other injury supervened.—*New York Medical Times.*

